# SANS serif: alignment-free, whole-genome-based phylogenetic reconstruction

**DOI:** 10.1093/bioinformatics/btab444

**Published:** 2021-06-16

**Authors:** Andreas Rempel, Roland Wittler

**Affiliations:** Genome Informatics, Faculty of Technology and Center for Biotechnology, Bielefeld University, 33615 Bielefeld, Germany; Bielefeld Institute for Bioinformatics Infrastructure (BIBI), Bielefeld University, 33615 Bielefeld, Germany; Graduate School “Digital Infrastructure for the Life Sciences” (DILS), Bielefeld University, 33615 Bielefeld, Germany; Genome Informatics, Faculty of Technology and Center for Biotechnology, Bielefeld University, 33615 Bielefeld, Germany; Bielefeld Institute for Bioinformatics Infrastructure (BIBI), Bielefeld University, 33615 Bielefeld, Germany

## Abstract

**Summary:**

SANS serif is a novel software for alignment-free, whole-genome-based phylogeny estimation that follows a pangenomic approach to efficiently calculate a set of splits in a phylogenetic tree or network.

**Availability and implementation:**

Implemented in C++ and supported on Linux, MacOS and Windows. The source code is freely available for download at https://gitlab.ub.uni-bielefeld.de/gi/sans.

**Supplementary information:**

Supplementary data are available at *Bioinformatics* online.

## 1 Introduction

In computational pangenomics and phylogenomics, a major challenge is the memory- and time-efficient analysis of multiple genomes in parallel. Conventional approaches for the reconstruction of phylogenetic trees are based on the alignment of specific sequences such as marker genes. However, the problem of multiple sequence alignment is complex and practically infeasible for large-scale data. Whole-genome approaches do neither require the identification of marker genes nor expensive alignments, but they usually perform a quadratic number of sequence comparisons (in terms of *k*-mers or other patterns) to obtain pairwise distances. This leads to a runtime that increases quadratically with the number of input sequences and is not suitable for projects comprising a large number of genomes.

We present the software SANS serif, which is based on a whole-genome approach and is both alignment- and reference-free. The command line tool accepts both assembled genomes and raw reads as input and calculates a set of splits that can be visualized as a phylogenetic tree or network using existing tools such as SplitsTree (Huson and Bryant, 2006). Instead of computing pairwise distances, the tool follows a pangenomic approach that does not require a quadratic number of sequence comparisons. The evaluation of our previous implementation SANS (Wittler, 2020) already showed promising results and revealed that our method is significantly faster and more memory-efficient than other whole-genome-based approaches. The new version SANS serif is a standalone re-implementation that does not rely on third-party libraries, introduces several new features and improves the performance of our method even further, reducing the runtime and memory usage to only ∼20% compared to the original implementation.

## 2 Features and approach

The general idea of our method is to determine the evolutionary relationship of a set of genomes based on the similarity of their whole sequences. Common sequence segments that are shared by a subset of genomes are used as an indicator that these genomes lie closer together in the phylogeny and should be separated from the set of all other genomes that do not share these segments. Each pair of such two sets forms a phylogenetic split, based on the concept of Bandelt and Dress (1992), and the lengths of the concerned segments contribute to the weight of the split, i.e. the length of the edge separating these two sets in the phylogeny.

**Fig. 1.**
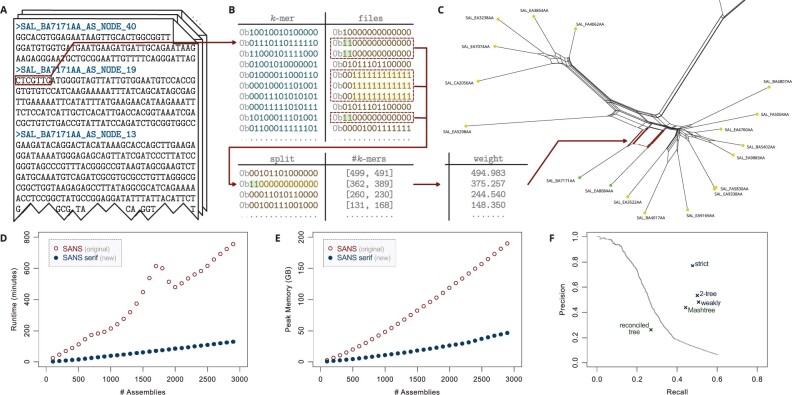
SANS serif methodology and evaluation. (**A**) One sequence file (FASTA/FASTQ) per genome/assembly is provided as input. For each file, the *k*-mers are extracted, transformed into bit vectors using a 2-bit encoding per character and stored in a hash table. (**B**) For each *k*-mer, another bit vector is stored to encode its presence or absence: a one (or zero) at position *i* indicates its presence (resp. absence) in the *i*th input file. Presence/absence patterns are combined to *splits* and stored in a second hash table together with two counts: the number of *k*-mers having that pattern and the number of *k*-mers having the complementary pattern. (**C**) Both counts are combined to an overall weight per split, e.g. using the geometric mean. Splits can be filtered and visualized as a network. As an example, a subnet of a weakly compatible split set of the *Salmonella* dataset (Zhou *et al.*, 2018) is shown. Splits are represented by parallel edges. (**D** and **E**) Runtime (user time) and peak memory usage of SANS on the *Salmonella* dataset on a single 2 GHz processor. For random subsamples of *n* assemblies and k=21, the 10*n* highest weighting splits were output. Values were averaged over processing three random subsamples each. (**F**) Accuracy w.r.t. the reference tree. Precision: (number of called splits also in the reference tree)/(number of all called splits), Recall: (number of reference splits also in the call set)/(number of all reference splits). Trivial splits, i.e. splits separating single leaves, are not considered. Filters: only considering the *x* highest weighting splits for increasing *x* (line), greedily extracting a subset that corresponds to a tree (strict), additionally keeping a second subset that corresponds to a tree (2-tree), greedily extracting a subset that is weakly compatible (weakly). For comparison, the accuracy of Mashtree (Katz *et al.*, 2019) with default parameters as well as a reconciled tree of 3002 core genes (Zhou *et al.*, 2018, Fig. 2A, supertree 2) is included, emphasizing the complexity of the dataset and the sensibility of the applied measure.

SANS serif accepts a list of multiple FASTA or FASTQ files containing complete genomes, assembled contigs or raw reads as input. In addition, the program offers the option to import a colored de Bruijn graph generated with the software Bifrost (Holley and Melsted, 2020). The lengths of the common sequences are counted in terms of *k*-mers, i.e. overlapping substrings of length *k*. The tool is capable of handling ambiguous IUPAC characters such as *N*’s, replacing these with the corresponding DNA bases, considering all possibilities. The output of the program is a tab-separated text file listing all calculated splits ordered by weight (or NEWICK format if applicable). Representing a phylogeny by a list of splits has the advantage that it allows to capture ambiguous signals in the input data that may arise, e.g. due to horizontal gene transfers and would be lost in a conventional phylogenetic tree. In these cases, the output corresponds to a phylogenetic network with the ambiguous signals appearing as parallel edges, as can be seen in Figure 1C. Several filter options allow to limit the output to a fixed number of most significant splits, to reduce the complexity of the network or to calculate a subset of the splits representing a tree.

## 3 Implementation

SANS serif is implemented in C++ and runs on all major platforms. The program can be compiled and executed without any dependencies and owes its good performance mainly to the way that the sequences are processed and stored in memory. The previous version of SANS used the Bifrost library (Holley and Melsted, 2020) to process the *k*-mers and construct a colored de Bruijn graph, from which the splits could subsequently be extracted. The new version SANS serif omits this rather time-consuming step of graph construction and is able to operate on the *k*-mers directly, leading to a significantly reduced runtime. Furthermore, the previous version used a trie data structure to store and process the splits, which required a large amount of memory for an increasing number of input files. In the new version, this data structure was replaced by two hash tables storing bit vectors, which showed to be much more memory efficient.

Each input file is processed by moving a window of a user-defined length *k* over the sequences and extracting the *k*-mers, as illustrated in Figure 1A. The program uses a hash table to store each *k*-mer as a key and a marker for the set of input files in which the *k*-mer is present as a value, updating the marker whenever the *k*-mer is observed in a new sequence. Both the *k*-mers and markers are encoded as bit vectors in order to reduce the amount of memory and enable fast bitwise comparisons and set operations. After all files have been processed, the table is scanned once to count the *k*-mers that belong to the same subset of input files. Each pair of complementary subsets is combined to one split, accumulating the corresponding two numbers of *k*-mers separately, as shown in Figure 1B. To this end, a second hash table is used with the split, represented by the bit vector of the smaller of the two subsets, as a key and the two *k*-mer numbers as a value. The overall weight of each split is calculated using the geometric mean of both numbers (optionally, by default, with pseudocounts, i.e. adding one each) and the selected filter is applied.

## 4 Results

Even though the new version complies with the same theoretical approach, the re-implementation improves both time and memory usage significantly. We evaluated the performance on a dataset comprising 2964 genomes of *Salmonella enterica* subspecies *enterica* (Zhou *et al.*, 2018). Figure 1D and E shows the runtime and memory usage of SANS serif compared to the original version of SANS depending on the number of input genomes. Supplementary Figures S1 and S2 additionally show the runtime and memory usage in dependence on the chosen *k*-mer length. The new version SANS serif manages to reduce the runtime and memory usage by ∼80%, processing the complete dataset on a single 2 GHz processor in almost 2 h using <50 GB of RAM (cf. Supplementary Table S1 for different parameter settings). Figure 1F shows the accuracy of the calculated split sets and filters. An earlier evaluation (Wittler, 2020) already revealed that our method has similar or better accuracy compared to other reference-free whole-genome-based approaches while demanding significantly less resources. Here, we also compare our program to another recent *k*-mer-based tool, Mashtree (Katz *et al.*, 2019). On the *S.enterica* dataset, Mashtree shows a slightly lower accuracy while requiring less memory and comparable runtime. However, since Mashtree—like almost all other phylogenetic reconstruction approaches—relies on pairwise distance computations, its runtime is dominated by a quadratic factor while SANS serif shows a linear trend in practice (cf. Supplementary Figure S3 for a more detailed comparison). To exemplify the ability to handle ambiguous IUPAC characters, we simulated a dataset of 100 genomes and incorporated *N*’s at random positions. The accuracy dropped significantly when the affected *k*-mers were skipped, whereas the new functionality to process these characters allowed to almost recover the original accuracy (cf. Supplementary Table S2).

## 5 Conclusion

SANS serif is a novel software that allows whole-genome-based phylogeny estimation with an unprecedented performance. A documentation listing all required and optional parameters as well as some quick start examples can be found on the project website.

## Supplementary Material

btab444_Supplementary_Data
