# Supplementary material for: SANS serif: alignment-free, whole-genome-based phylogenetic reconstruction
Source: Bioinformatics. 2021 Jun 16;37(24):4868–70. doi: 10.1093/bioinformatics/btab444 (PMC8665756; doi:10.1093/bioinformatics/btab444)
Supplement: btab444_Supplementary_Data [file btab444_supplementary_data.zip › supplements.pdf]

SANS serif: alignment-free, whole-genome based phylogenetic reconstruction

Andreas Rempel [1,2,3] and Roland Wittler [1,2]

[1]Genome Informatics, Faculty of Technology and Center for Biotechnology, Bielefeld University, 33615 Bielefeld, Germany,
[2]Bielefeld Institute for Bioinformatics Infrastructure (BIBI), Bielefeld University, 33615 Bielefeld, Germany, and
[3]Graduate School “Digital Infrastructure for the Life Sciences” (DILS), Bielefeld University, 33615 Bielefeld, Germany.

Supplementary Material

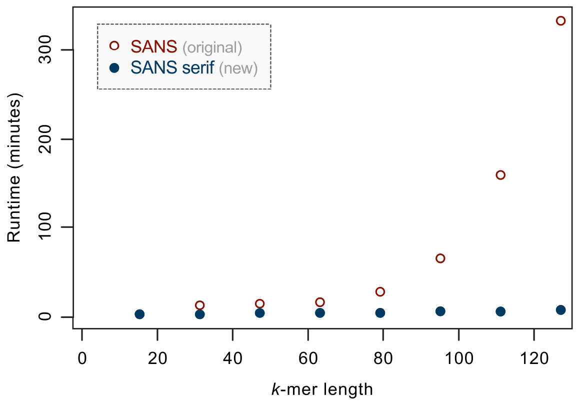

Fig. 1. Runtime (user time) of SANS evaluated for different k-mer lengths. For random subsamples of 100 assemblies from the *Salmonella* data set, the 1000 highest weighting splits were output. Values were averaged over processing three random subsamples each.

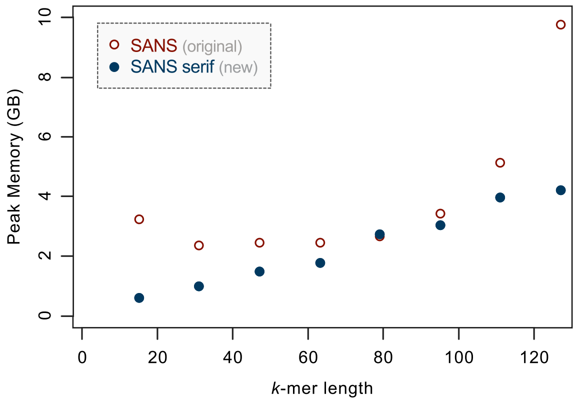

Fig. 2. Peak memory usage of SANS evaluated for different k-mer lengths. For random subsamples of 100 assemblies from the *Salmonella* data set, the 1000 highest weighting splits were output. Values were averaged over processing three random subsamples each.

Table 1. Runtime (user time) and peak memory usage of SANS serif on the complete *Salmonella enterica* data set evaluated for different parameters: processing the sequence files directly (−input), processing a colored de Bruijn graph generated from the sequences using Bifrost (−graph), processing a list of splits and applying different filters (−filter), and processing the sequence files considering IUPAC characters (−iupac). The overall weight of each split was calculated using the geometric mean without pseudocounts (−m geom). Values were averaged over three different runs each.

Parameter		Runtime (min)	Memory (GB)
−input		132.71	47.57
−graph		69.08	51.28
−filter	*strict*	0.06	0.01
	2-tree	0.06	0.01
	weakly	467.89	0.01
−iupac		153.29	47.59

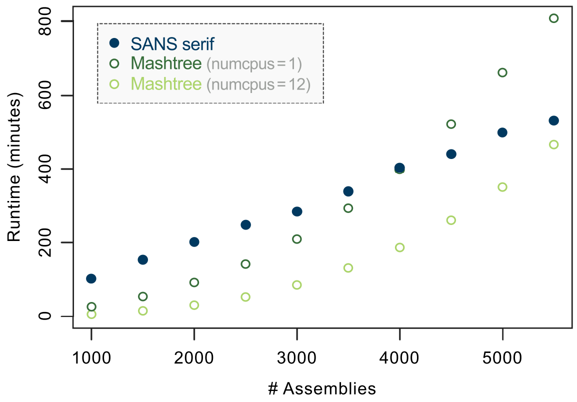

Fig. 3. Runtime (real time) comparison of SANS serif and Mashtree (Katz *et al.*, 2019) on *Salmonella* Typhimurium assemblies. For 6000 randomly selected genomes from a total of 19 237 that were annotated as serovar *Typhimurium* in EnteroBase (Alikhan *et al.*, 2018) (on April 09, 2019), 5712 assemblies were available and have been downloaded. For random subsamples of n assemblies, both Mashtree and SANS serif have been run with k=21 (Mashtree default). For SANS serif, the $10n$ highest weighting splits were determined using the geometric mean and filtered for a tree. Mashtree has been run with default parameters as well as using multi-threading (numcpus=12). Values were averaged over processing two random subsamples each. The peak memory usage of SANS with 29.9 GB was higher than that of Mashtree with 9.3 GB for processing 5500 assemblies (data not shown). The runtime of Mashtree shows a quadratic increase (least squares regression, adjusted R-squared 0.9998), whereas SANS serif shows a linear trend (adjusted R-squared 0.9975).

Table 2. Effect of `N`'s in the input sequences. A phylogeny with 100 leaf genomes of length ∼96 kb and an evolutionary distance of 5 PAM to the root has been simulated with ALF (Dalquen *et al.*, 2012). (See software repository for the complete parameter setting.) DNA characters in the genomes were randomly substituted by `N`'s at a rate of 0.1 % and the sequences were processed by SANS serif before and after the substitution with k-mers containing `N`'s being skipped (default) or `N`'s replaced by all possible bases (parameter −iupac, x=16). For the obtained tree-filtered split sets, precision and recall (cf. Figure 1) as well as weighted precision (total weight of called splits also in the reference tree) / (total weight of all called splits) and weighted recall (total weight of reference splits also in the call set) / (total weight of all reference splits) have been computed.

	original	with 0.1 % N's	
		skipped	replaced
unweighted			
precision	0.90	0.63	0.78
recall	0.70	0.38	0.58
weighted			
precision	0.98	0.95	0.98
recall	0.88	0.68	0.88

References

Alikhan, N.F. *et al.* (2018) A genomic overview of the population structure of *Salmonella*, *PLoS Genet.*, **14(4)**, 1007261.

Dalquen, D.A. *et al.* (2012) ALF – A simulation framework for genome evolution *Mol. Biol. Evol.*, **29(4)**, 1115–1123.

Katz, L. *et al.* (2019) Mashtree: a rapid comparison of whole genome sequence files, *J. Open Source Software*, **4(44)**, 1762.
